# Comparison of Long-Term Outcomes of Living-Donor Liver Transplantation in Hepatocellular Carcinoma Based on the Milan Criteria After Downstaging According to Degradable Starch Microspheres with Transcatheter Arterial Chemoembolization

**DOI:** 10.3390/life16091532

**Published:** 2026-09-15

**Authors:** Umut Tüysüz, Tonguc Utku Yılmaz, Imam Bakır Batı, Işıl Yıldız

**Affiliations:** 1 Department of General Surgery, Sarıyer Hamidiye EtfalTraining and Research Hospital, Istanbul 34453, Turkey; 2 Department of General Surgery, School of Medicine, Acibadem Mehmet Ali Aydinlar University, Istanbul 34303, Turkey; 3 Department of Liver transplantation, Bursa Acıbadem Hospital, Bursa 16210, Turkey; 4 Vocational School of Health Services, Medical İmaging Techniques, Acibadem Mehmet Ali Aydinlar University, Istanbul 34303,Turkey

**Keywords:** living-donor liver transplantation, hepatocellular carcinoma, downstaging therapy, prognosis

## Abstract

Objectives: Hepatocellular carcinoma (HCC) is a disease with an increasing incidence. Liver transplantation (LT) represents the sole curative treatment option. Downstaging (DS) therapy represents an option in selecting suitable candidates for transplantation who initially have tumors exceeding the MC. Consequently, patients undergoing DS for MC have demonstrated comparable survival rates to those who initially met the MC. Degradable starch microspheres with transcatheter arterial chemoembolization (DSM-TACE), a type of TACE, have also been recently introduced in clinical practice. Methods: This retrospective study encompassed 200 consecutive adult patients who underwent living-donor LT for HCC and cirrhosis at two different centers from March 2012 to December 2023. We aimed to compare long-term overall survival (OS) and recurrence-free survival (RFS) among patients with HCC who met the DSM-TACE criteria for MC following diagnosis, those initially within MC, and those outside MC following diagnosis. Results: The OS rate in the MC group was statistically comparable to that in the DS and beyond the MC groups. The MC and DS groups exhibited comparable long-term RFS rates. However, the MC and DS groups showed significantly better RFS rates than the beyond the MC group. Microvascular invasion (MVI), tumor size and number, and the beyond the MC group were identified as significant recurrence risk factors, whereas MVI alone was an independent prognostic factor. Conclusions: Our results showed good long-term OS and RFS in HCC patients following LDLT after staging into MC with DSM-TACE. Additionally, we achieved a more acceptable survival rate compared to other treatments in the group that underwent MC staging after DS.

## 1. Introduction

Hepatocellular carcinoma (HCC) is the third leading cause of cancer-related mortalities globally. Its prevalence rate is anticipated to progressively increase [1]. Liver transplantation (LT) is the gold standard in the management of underlying liver cirrhosis and tumors [2]. The increase in HCC-related mortality and incidence has led to continuous improvements in patient selection policies. The Milan criteria (MC) represent the first global standard that offers excellent post-transplant survival outcomes by establishing upper limits for tumor burden. The MC has established a standardization of suitable patients for transplantation (a single lesion of ≤5 cm in size or 2–3 lesions of ≤3 cm in size) [3]. Recently, high cure rates have been reported in patients with HCC undergoing LT. This promising outcome may be attributed to the use of the MC, which encompasses the morphological characteristics of the tumor, in strict pretransplant selection. However, approximately 70% of patients with HCC are diagnosed at an advanced stage of the disease, preventing curative interventions. Besides using broad selection criteria to increase the number of recipients who can achieve acceptable post-LT outcomes, downstaging (DS) with locoregional therapy (LRT) represents another option [3]. Currently, DS is an option in selecting suitable candidates for transplantation who initially have tumors exceeding the MC. Initial results have demonstrated favorable outcomes with recurrence-free survival (RFS) rates of 87%. However, the recurrence rate, reflecting poor prognosis following LT, remains between 8% and 20% [4]. A large cohort study revealed that approximately 65% of patients with HCC were patients in the beyond the MC group. Of these patients, 42% underwent LRT before transplantation [5,6]. However, no studies have employed prognostic markers for predicting the increased risk of recurrence following transplantation in this specific patient group. Furthermore, treatment outcomes vary owing to heterogeneity in tumor biology and liver function.

Only 22% of patients with recurrence survive for >5 years. Therefore, predicting the risk of post-LT recurrence is clinically significant in donor selection strategy [7]. DS treatments comprise transarterial interventions, including transcatheter arterial chemoembolization (TACE) and TARE, and ablative methods, including percutaneous ethanol injection, radiofrequency ablation, and microwave ablation [8]. Within the broad criteria, the use of DS management is largely based on response to LRT. Consequently, a subgroup patient selection strategy beyond the MC, considered to have more suitable tumor biology for LT, has been increasingly employed [9]. However, recently, reported success rates following DS vary owing to the variability of follow-up periods and practices with different cutoff values for baseline tumor burden. The previous meta-analysis was inadequate in interpreting results on the basis of the baseline criterion of tumor burden due to the scarcity of data in a specific period for DS [10]. The American Association for the Study of Liver Diseases guidelines recommend LT following successful DS within the MC for patients beyond the MC. However, the level of evidence and the strength of this recommendation are low [11]. Most studies have considered tumor regression meeting MC based on morphological features as successful DS. However, data on LT outcomes following DS remain inconsistent and predominantly based on uncontrolled studies [3]. No consensus exists regarding the minimum observation period from DS to LT, survival outcomes following DS LT, standard endpoints for DS treatment, eligibility criteria for LT, and appropriate patient selection for DS [12]. The effectiveness of DS treatment relies on common markers comparable to those in patients initially within the MC and undergoing LT. These markers encompass the presence of histological markers, including microvascular invasion (MVI), and the absence of satellite lesions, as well as low-grade tumors, which indicate good prognosis in HCC. Compared with other LRT methods, TACE is a very well-standardized procedure. The microspheres used in this method can cause irreversible ischemia and tumor tissue necrosis. Consequently, vascular endothelial growth factor (VEGF) decreases. This reduction negatively impacts the growth and metastatic ability of the tumor [13]. Recently, degradable starch microspheres with transcatheter arterial chemoembolization (DSM-TACE) have become broadly employed in clinical practice for DS in HCC [14]. Starch microspheres cause temporary arterial occlusion lasting up to 50 min before being degraded by serum alpha-amylase. During this time, increased intratumoral concentration of the cytostatic agent is achieved. The temporary obstruction reduces the stimulation of hypoxia-sensitive factors and the release of VEGF, which induces neoangiogenesis, tumor proliferation, and metastatic growth. This action makes DSM-TACE a robust option among therapeutic strategies targeting the tumor stromal microenvironment when combined with conventional chemotherapeutic agents [15]. This study primarily aimed to compare long-term overall survival (OS) and RFS and, secondarily, tumor recurrence risk factors among patients with HCC who met the DSM-TACE (EmboLog^®^ S; Serumwerk Bernburg AG, Bernburg, Germany)and MC following DS and were initially within the MC but were beyond the MC following DS and underwent living-donor LT (LDLT).

## 2. Materials and Methods

This retrospective study included 200 consecutive adult patients who underwent LDLT for HCC and cirrhosis at two different centers from March 2012 to December 2023. The following were the inclusion criteria: patients with pathology results showing HCC, those undergoing LRT, those initially meeting MC, those undergoing first-time LT for HCC, and those receiving LDLT. The following were the exclusion criteria: patients who had previously undergone LT, those with pathology results demonstrating no cancer or intrahepatic cholangiocarcinoma or mixed-type HCC, those with incidental HCC, those undergoing cadaveric LT, those with metastatic liver cancer, those with pre-LT extrahepatic disease and macrovascular invasion, those with recurrent HCC, those who did not meet the MC initially based on specimen examination and not receiving LRT, patients who had any early mortality unrelated to HCC within the first 30 days following LT. The patients must not have had any chemotherapeutic or immunotherapeutic regimen before LT.

**Figure 1:**
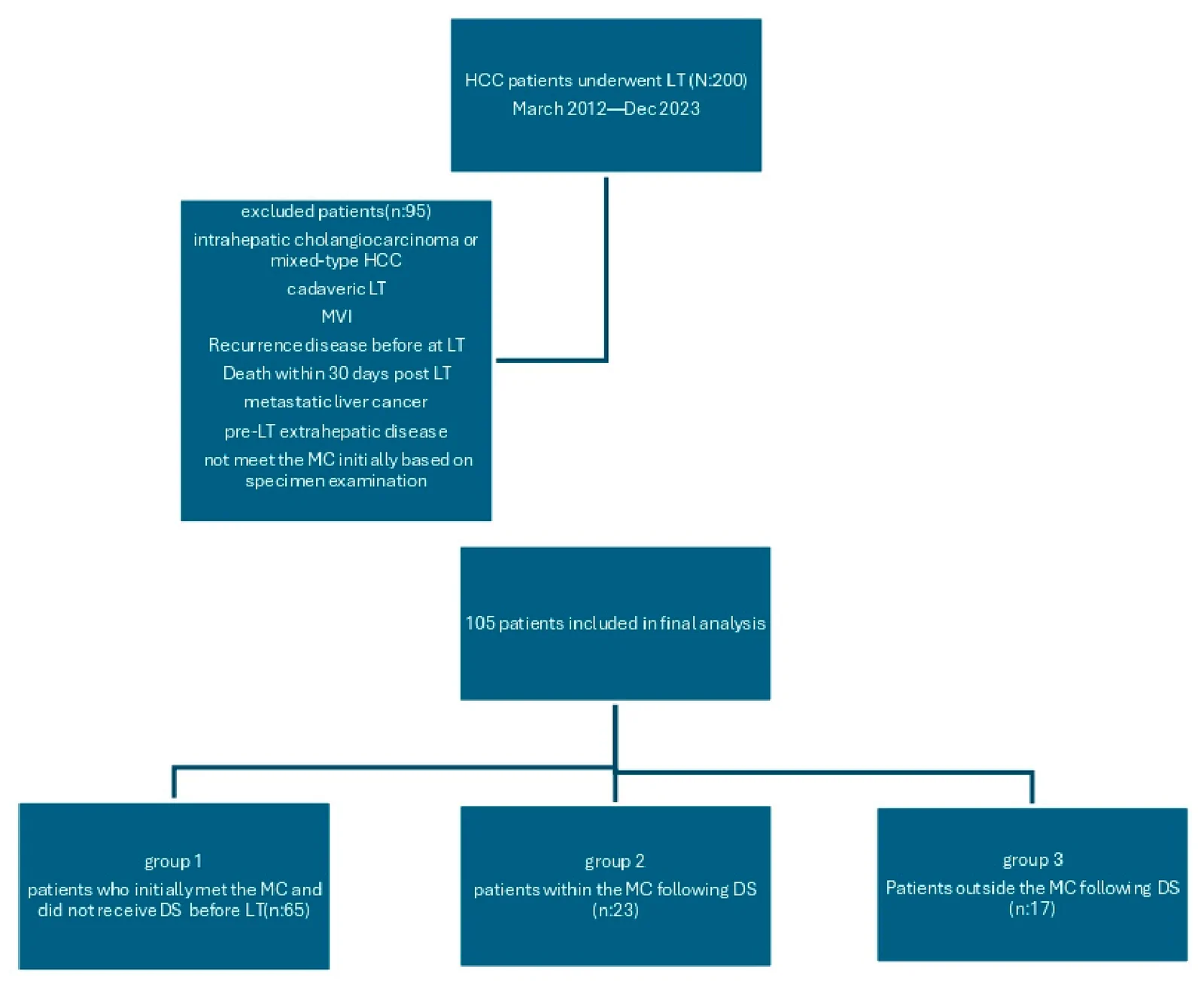
Flowchart illustrating study populations.

After applying these criteria, 105 patients with HCC were included in the final analysis. Of these patients, 65 (61.9%) were within the MC before transplantation, 23 (21.9%) were within the MC following DS, and 17 (16.2%) were patients who responded to treatment following DS but were outside the MC group. Post-LT results in the DS group were compared with those of the patients who initially met the MC. Patients who progressed beyond the MC or who could not be included in the MC with DS treatment but eventually underwent LT were categorized as the beyond the MC group (Figure 1). The two centers in this study had comparable practical and clinical patterns, LT recipient population, waiting time, and experience. Successful tumor DS was defined as a reduction in the viable tumor burden within the MC following LRT. A minimum 6-week observation period was applied before transplantation following DS. All patients underwent magnetic resonance imaging (MRI) at 4 weeks post-treatment (DSM-TACE) to assess tumor response according to the modified Response Evaluation Criteria in Solid Tumors (mRECIST) criteria and determine if another treatment season was required. Complete response was defined as the complete disappearance of intratumoral arterial contrast enhancement in all target lesions. Partial response was defined as at least a 30% decrease in the total diameter of viable (contrast-enhancing) target lesions. Progressive disease was defined as at least a 20% increase in the total diameter of viable (contrast-enhancing) target lesions and encompassed all cases not classified as stable disease, partial response, or progressive disease [16]. Tumor burden was estimated using MRI according to standard protocols. The analysis included the number of viable tumors and the maximum tumor diameter determined by imaging before LRT. Patients were classified according to the pathological examination results of the LT specimens taken following LRT treatment, and the analysis was performed accordingly. We did not set an upper limit in terms of the period between two treatments and the number and size of tumors with LRT repetition. Post-LRT follow-up protocol was performed at 3–6-month intervals with imaging and alpha-fetoprotein (AFP) measurement. Recurrence diagnosis was based on imaging and AFP elevation. Pathological examination was performed for confirmation when necessary. Our immunosuppressive treatment protocol includes a calcineurin inhibitor (CNI), mycophenolate mofetil (MMF), and corticosteroids. We initiate prednisolone at a dose of 500 mg intravenously during transplantation; the dosage is tapered from 100 mg to 20 mg within the first eight postoperative days and reduced to 5 mg by the second month before being discontinued. We add MMF to the regimen when a reduction in the CNI dose is required due to renal dysfunction.

### Statistical Methods

Categorical variables were summarized as number and percentage, and continuous variables as median and interquartile range (IQR). Overall survival (OS) was measured from transplantation to death from any cause. The endpoint termed recurrence-free survival (RFS) in this study was defined as the interval from transplantation to the first documented HCC recurrence; patients without documented recurrence were censored at death or last follow-up. Survival functions were estimated using the Kaplan–Meier method. The three Milan-criteria (MC) groups were compared using the global log-rank test, and pairwise log-rank comparisons were interpreted after Holm adjustment. Median follow-up was estimated using the reverse Kaplan–Meier method.

Univariable associations with OS and RFS were evaluated using conventional Cox proportional-hazards regression with the Efron approximation for tied event times. Because only 16 recurrences and 24 deaths occurred, multivariable models containing all candidate covariates would have been severely overparameterized. Multivariable analyses were therefore limited to the principal study exposure, MC category (2 degrees of freedom), and microvascular invasion (MVI; 1 degree of freedom), an established pathological prognostic factor that is not a component of the MC. Tumor size and lesion number were not entered together because they overlap conceptually with MC classification; AFP, MELD, and Child–Pugh class were examined only in univariable analyses. No stepwise variable selection was used.

Multivariable models were fitted using Firth’s bias-reduced penalized partial-likelihood Cox regression with Breslow handling of ties Hazard ratios (HRs) are reported with 95% profile penalized-likelihood confidence intervals (CIs), and *p*-values were derived from penalized likelihood-ratio tests. Initially within the MC and absence of MVI were the reference categories. For multilevel categorical variables, overall factor *p*-values and level-specific comparisons with the reference category are reported separately. Proportional-hazards assumptions were evaluated in the corresponding conventional Efron-ties Cox models. Model coefficients and Schoenfeld residuals were obtained with statsmodels; the residuals were scaled and the Grambsch–Therneau test statistic was calculated using the Kaplan–Meier time transformation in a self-contained implementation. A rule-based sensitivity analysis re-derived RFS timing from recorded recurrence and follow-up information.

Descriptive summaries and Kaplan–Meier/global log-rank analyses were performed in SPSS for Windows, version 15.0 (SPSS Inc., Chicago, IL, USA) and independently reproduced. The reported univariable Cox estimates, reverse Kaplan–Meier follow-up estimate, Holm-adjusted pairwise log-rank tests, Firth-penalized Cox models, profile CIs, penalized likelihood-ratio tests, proportional-hazards diagnostics, timing sensitivity analysis, and figures were generated in Python 3.12.3. Conventional Efron-ties Cox models and Schoenfeld residuals were obtained with statsmodels 0.14.6. Proportional-hazards test *p*-values were calculated with an archived self-contained implementation of the Grambsch–Therneau test using the Kaplan–Meier time transform, matching the algorithm used by lifelines 0.30.3. Firth estimation and profile-likelihood procedures were implemented with NumPy 2.4.4 and SciPy 1.17.1 following Heinze and Schemper. All tests were two-sided, and *p* < 0.05 was considered statistically significant.

## 3. Results

**Figure 2:**
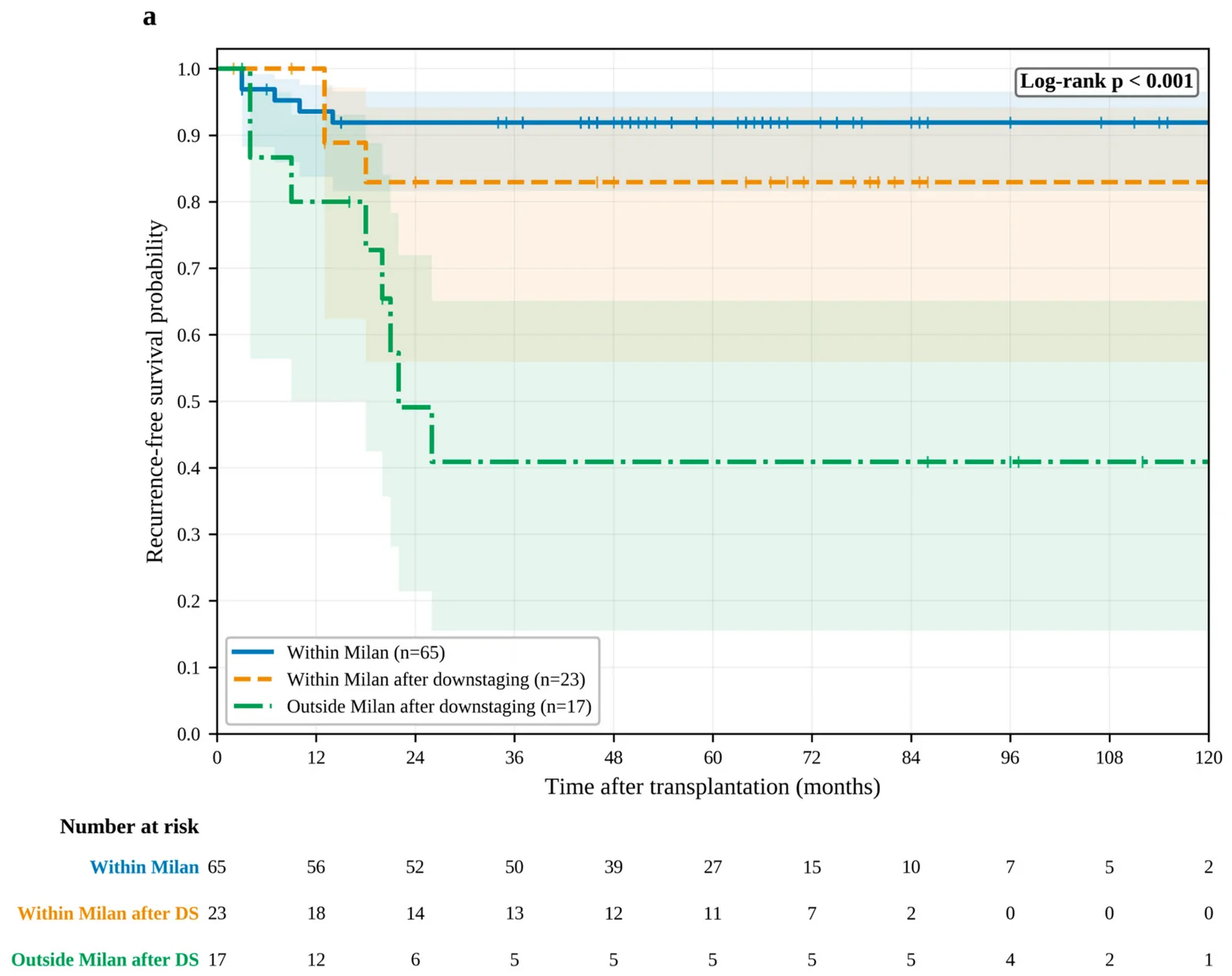

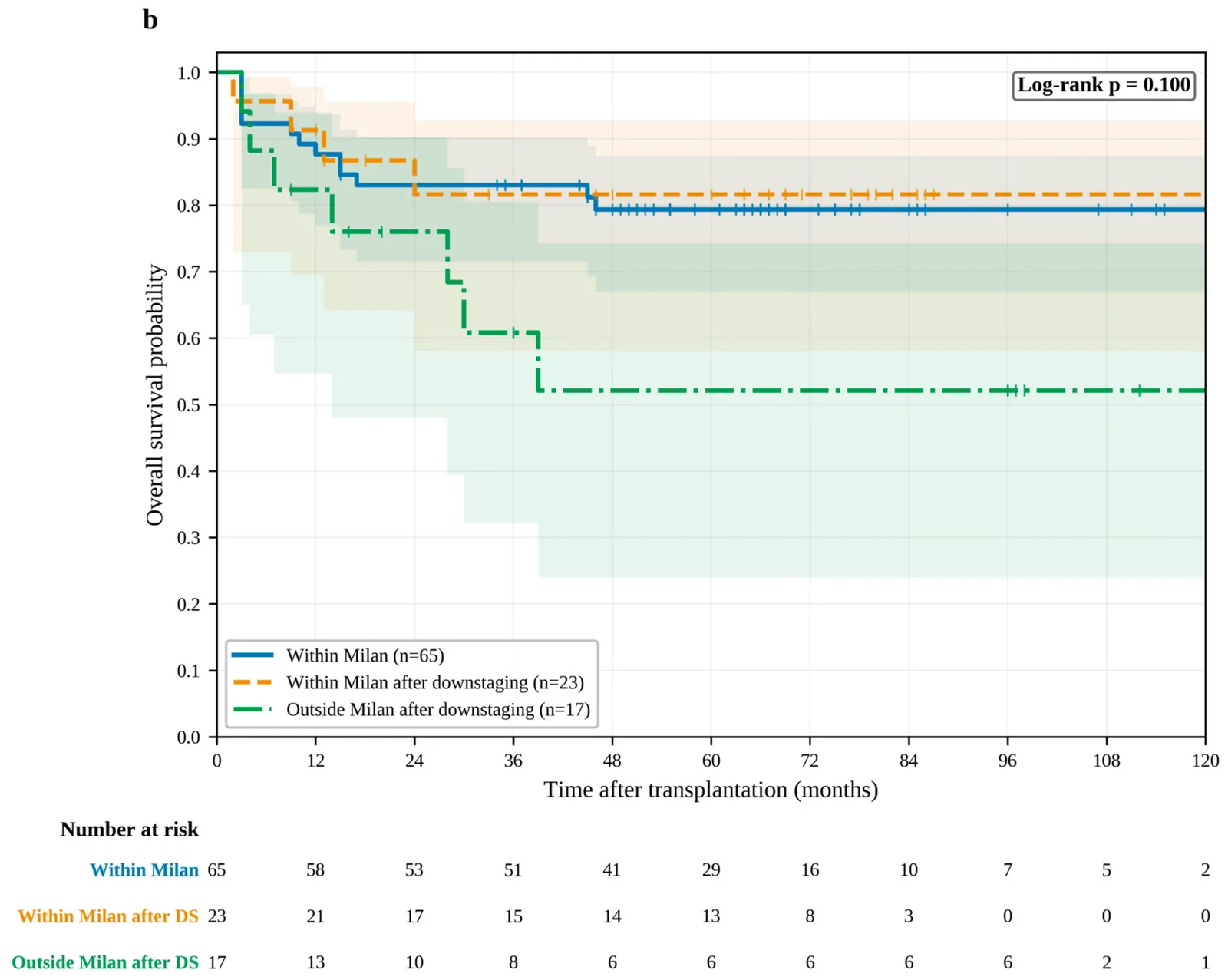
(**a**) Recurrence-free survival by Milan category. Global log-rank *p* < 0.001. Solid, dashed, and dash-dot lines permit grayscale discrimination. (**b**) Overall survival by Milan category. Global log-rank *p* = 0.100. Solid, dashed, and dash-dot lines permit grayscale discrimination.

The demographic and clinicopathological characteristics of the study population are presented in Table 1. Overall, 105 patients were included in this study. Of these patients, 83 (79.0%) were male, and 22 (21.0%) were female. The median age was 66.9 years. The most common etiological cause was HBV infection (*n* = 58 [55.2%]), followed by cryptogenic cirrhosis 16 (15.2%) and HCV infection 14 (13.3%). Regarding liver function status, 58 (55.2%), 40 (38.1%), and seven (6.7%) patients were classified as Child–Pugh A, B, and C, respectively. Forty patients (38.1%) had undergone LRT. Forty patients (38.1%) who received only DSM-TACE as DS therapy were included in this study, whereas sixty-five patients (61.9%) had not received DS therapy. Among patients evaluated for response to LRT, the most frequent outcome was regression (*n* = 23 [57.5%]); stable disease, progression, and complete response were observed in 12 (30.0%), three (7.5%), and two (5.0%) patients, respectively. The median observed follow-up period was 55 months. Tumor recurrence was observed in 16 patients (15.2%) and 24 (22.9%) died during follow-up. In the total cohort, 1-, 3-, and 5-year RFS rates were 92.7%, 82.5%, and 82.5%, respectively. When evaluated according to the MC, the 1-, 3-, and 5-year RFS rates for the within Milan group at baseline were 93.6%, 91.9%, and 91.9%, respectively. The beyond Milan following DS group exhibited a significantly lower RFS, with 1-, 3-, and 5-year RFS rates of 80.0%, 40.9%, and 40.9%, respectively. In the within Milan following DS group, 1-, 3-, and 5-year RFS rates were 100%, 83.0%, and 83.0%, respectively. Median RFS was reached only in the beyond-MC group (22 months) and was not reached in the other two groups. Recurrences occurred at a median of 13 months after transplantation and all 16 recurrences occurred within 26 months. RFS differed significantly among the three MC groups (global log-rank *p* < 0.001). Subgroup comparisons revealed that the beyond Milan following DS group demonstrated significantly lower RFS rates than the within Milan group. Moreover, a significant difference was observed between the beyond Milan following DS and within Milan following DS groups. However, no significant difference in RFS was noted between the within Milan and within Milan following DS groups (Figure 2a).

As shown in Figure 2b, regarding OS, 1-, 3-, and 5-year survival rates were 87.6%, 79.6%, and 76.0%, respectively.

In the subgroup analysis based on the MC, 1-, 3-, and 5-year survival rates were 87.7%, 83.0%, and 79.4%, respectively, for the initially within Milan group. For the beyond Milan following DS group, 1-, 3- and 5-year OS rates were 82.4%, 60.8%, and 52.1%, respectively. For the within Milan following DS group, these rates were 91.3%, 81.6%, and 81.6%, respectively.

Although some numerical differences were noted for OS between the groups, the global log-rank comparison was not statistically significant. No evaluated variable was significantly associated with OS in univariable Cox analyses (Table 2). In the Firth-penalized multivariable model, MVI was not independently associated with mortality (adjusted HR 1.462, 95% profile CI 0.624–3.355; *p* = 0.375). Relative to patients initially within the MC, the adjusted HR was 2.214 (95% profile CI 0.818–5.603; *p* = 0.113) for patients beyond the MC after downstaging and 0.959 (95% profile CI 0.290–2.607; *p* = 0.938) for patients within the MC after downstaging. The overall adjusted MC-category effect was not significant.

The beyond Milan following DS group demonstrated a significantly higher risk of recurrence than the reference group (patients initially within the MC). By contrast, the risk of recurrence in the within Milan following DS group did not significantly differ from that of the initially within Milan group.

In univariable Cox analyses, MVI, maximum tumor size, number of HCC lesions, and MC category were associated with RFS (Table 3). MVI was associated with a 9.641-fold recurrence hazard (95% CI 2.746–33,848; *p* < 0.001). Each 1 mm increase in maximum tumor size was associated with HR 1.030 (95% CI 1.009–1.052; *p* = 0.006), and each additional lesion with HR 1.147 (95% CI 1.021–1.287; *p* = 0.020). The overall univariable MC-category effect was significant (*p* = 0.002).

**Figure 3:**
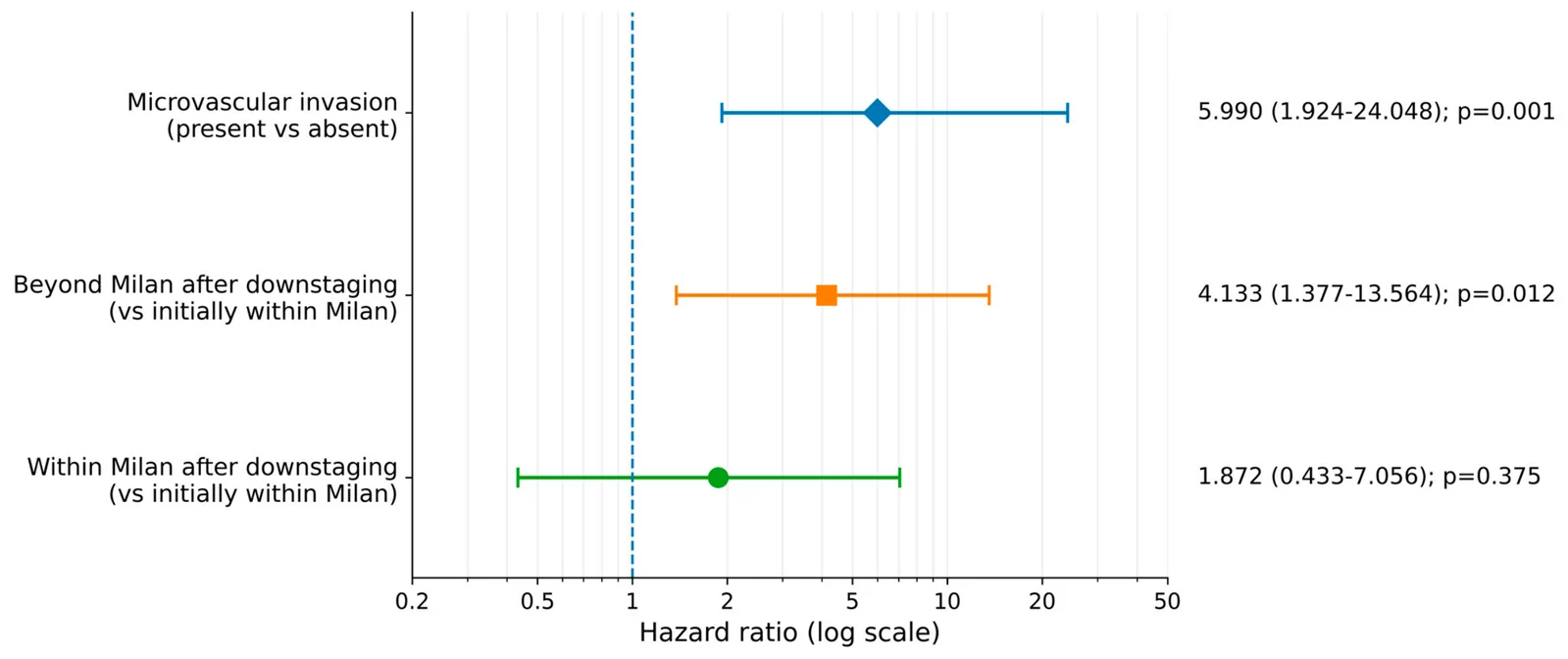
Firth-penalized multivariable Cox model for recurrence-free survival; adjusted HRs with 95% profile penalized-likelihood CIs.

In the Firth-penalized multivariable model, MVI remained associated with higher recurrence hazard (adjusted HR 5.990, 95% profile CI 1.924–24,048; *p* = 0.001) (Figure 3). Relative to patients initially within the MC, patients beyond the MC after downstaging also had higher recurrence hazard (adjusted HR 4.133, 95% profile CI 1.377–13,564; *p* = 0.012), whereas patients within the MC after downstaging did not differ significantly from the reference group (adjusted HR 1.872, 95% profile CI 0.433–7.056; *p* = 0.375). The overall adjusted effect of the MC category was significant (penalized likelihood-ratio *p* = 0.041).

Scaled Schoenfeld-residual tests supported proportional hazards for MVI (*p* = 0.724) and the within MC after downstaging contrast (*p* = 0.126), but indicated time dependence for the beyond MC after downstaging contrast in the RFS model (*p* = 0.021). The corresponding HR should therefore be interpreted as a follow-up-weighted average over the observed early-recurrence period rather than as a constant effect throughout long-term follow-up. No proportional-hazards violation was detected in the OS model (MVI *p* = 0.556; beyond MC after downstaging *p* = 0.513; within MC after downstaging *p* = 0.612). A timing sensitivity analysis based on re-derived RFS durations for nine patients allowed all 105 patients to contribute positive follow-up and produced results consistent with the primary analysis: MVI HR 6.172 (95% profile CI 1.994–24,696; *p* = 0.001), beyond MC after downstaging HR 4.145 (95% profile CI 1.386–13,544; *p* = 0.011), within MC after downstaging HR 1.712 (95% profile CI 0.396–6.458; *p* = 0.445), overall MC-category *p* = 0.040, and global log-rank *p* < 0.001.

## 4. Discussion

In this study, we aimed to overcome the unmet need through a retrospective analysis of 40 patients diagnosed with HCC beyond the MC and who underwent LRT (DSM-TACE) for DS before LDLT. For the first time, we compared two subgroups of LDLT recipients who initially had HCC within the MC with two subgroups of patients who were downstaged into MC following DSM-TACE as LRT and who remained beyond the MC following DS. The principal finding of this study is that post-transplant recurrence risk differed according to Milan status after downstaging, whereas OS did not differ significantly among the three groups. Patients downstaged to within the MC had long-term RFS and OS estimates that did not differ significantly from those of patients initially within the MC. In contrast, patients who remained beyond the MC after downstaging had substantially lower RFS. HCC recurrence represents the most significant predictor of mortality in patients undergoing LT. Identifying high-risk patients before LT highlights the significance of patient stratification [17]. A study comparing patients within the MC to those meeting the UNOS-DS criteria reported comparable 3-year survival rates between the two groups (83% and 79%, respectively). However, patients with tumor burdens exceeding the UNOS-DS criteria exhibited a lower survival rate (71%) [18]. The major finding was that the group falling within the DS criteria demonstrated satisfactory long-term recipient OS and RFS rates. These rates were comparable to those of the group initially within the MC. Although extrahepatic tumor spread and macrovascular invasion are contraindications for DS, a recent systematic review has not established an upper limit for tumor burden based on tumor size and number. Here, no significant association was found between post-LT recurrence and the time spent on the waiting list [19]. Similarly, our center does not employ a restrictive limit for DS. Currently, no universally accepted guidelines regarding DS eligibility criteria or optimal DS protocols exist; although, MVI, maximum tumor size, and tumor number were identified as significant factors for RFS in the univariate analysis. A nine-coefficient recurrence model would provide only approximately 1.8 events per coefficient and would be vulnerable to overfitting and sparse-data bias. Limiting the analysis to three model degrees of freedom and applying Firth penalization yielded more stable estimates for the clinically prioritized effects. In this model, both MVI and remaining beyond the MC after downstaging were associated with recurrence. These estimates remain exploratory because the confidence intervals are wide.

The most critical determinant in this study was HCC recurrence, highlighting the significance of classifying this patient group before LT. Although findings regarding risk factors impacting post-LT survival and recurrence in downstaged HCC have recently emerged, our results aligned with previous studies and confirmed associations with poor prognostic factors following LT [20]. Studies on patients undergoing DS have analyzed strict inclusion criteria and mandatory pre-LT waiting periods, reporting recurrence and survival rates comparable to those of patients who initially met the MC [21]. Although several studies investigating LT outcomes following DS reported excellent 1-year survival rates (90%), 5-year OS results varied (70–90%). One- and five-year RFS rates were 80% and 91%, respectively [22]. Targeting the MC as a cutoff in successful responses to LRT can be used in selecting a patient group with a good prognosis. Furthermore, we have demonstrated that a successful tumor response to DSM-TACE, downstaging the tumor within the MC, predicts superior OS and RFS. Our analysis revealed that, regardless of the initial tumor stage, a 6-week waiting period was satisfactory for evaluating the tumor response to LRT and the biological characteristics of the tumor, as well as for identifying patients at low risk for poor post-transplant outcomes associated with tumor progression. However prospective studies comparing observation intervals and including patients who do not proceed to transplantation are needed to address this question.

A favorable tumor response to LRT can also function as a parameter for predicting positive prognostic indicators, including low tumor grade and the absence of microvascular or lymphatic invasion, which may otherwise remain undetected without a pretransplant biopsy. The favorable outcomes observed among patients downstaged to within the MC are consistent with the response-based selection concept reported in transplant and downstaging cohorts. Previous studies have indicated that the degree of tumor necrosis following pretransplant LRT was correlated with a reduced risk of recurrence and enhanced survival [23]. Patients who achieved a pathological complete response following treatment demonstrated survival and recurrence rates comparable to those of patients whose tumors initially met the MC [24]. However, although mRECIST is the most broadly employed method for assessing tumor response to treatment, pathological examination of the explant offers a more precise assessment of the tumor’s biological characteristics and the extent of necrosis. Maintaining the radiological response achieved through DS is of greater relevance in the pretransplant management phase. Furthermore, the observation that the radiological response to treatment was disproportionately higher than the pathological response, aligning with findings of other studies, led us to adopt the pathological response as a criterion [25]. Consistent with this finding, the present study confirmed an association between tumor necrosis achieved via DSM-TACE (used as a DS method before LT) and lower HCC recurrence and improved survival. Our study demonstrated long-term outcomes and examined the role of DS. However, it did not evaluate intention-to-treat (ITT) results or the treatment of recurrence. To predict survival outcomes in patients with HCC who underwent LT following DS, we conducted a pooled analysis using individual participant data. Overall, >50% of the patients included in this study successfully underwent DS. Our results demonstrated that patients who underwent successful DS from a state exceeding the MC exhibited post-LT survival and RFS rates comparable to those of patients who were within the MC at baseline. However patients who remain beyond the MC despite response represent a higher-recurrence subgroup. This group may warrant particularly close surveillance during the first two post-transplant years, when all recurrences in this cohort occurred.

This finding may be associated with the neoadjuvant effect of the DS procedure. Furthermore, less stringent selection criteria for DS before LT may predispose patients to a higher risk of recurrence. In a study utilizing the MC as the benchmark for DS, only patients who were successfully downstaged to within the criteria underwent orthotropic LT. In the DS group, the 3- and 5-year post-transplant OS rates were 88.2% and 73.5%, respectively, whereas the 3- and 5-year post-transplant RFS rates were 82.8% and 62.1%, respectively [26]. A previous study reported that for patients who were initially beyond the MC, successful DS to within the criteria led to 5-year post-LT survival rates comparable to those of patients who were within the MC at baseline [27]. Considering the scarcity of available donor livers and the ongoing uncertainty regarding the selection of patients who would derive the greatest benefit from LT, the optimal management of patients with HCC exceeding the MC remains controversial. Results from most studies utilizing the MC for DS criteria were inconclusive owing to short follow-up periods, the absence of comparison groups, and small sample sizes [28]. Moreover, whether LT is indicated for patients who remain beyond the MC following DS therapy remains debated. Data regarding long-term OS and RFS in this patient population are scarce. To date, no studies have predicted recurrence risk and survival following LDLT in patients with HCC exceeding the MC who underwent DEB-TACE as a DS method. Predicting recurrence and survival following LDLT in patients who remain beyond the MC following LRT is crucial for clinical practice and optimizing donor selection. Recent LT criteria have been based on tumor number and size, pre-operative AFP levels, tumor response to DS therapy, tumor differentiation, and the presence of macrovascular invasion [29]. In the present study, we investigated tumor necrosis rates, sex, age, pre-TACE AFP, MELD score, underlying liver disease, Child–Pugh class, maximum tumor diameter, tumor number, MVI, graft-to-recipient weight ratio, and cold ischemia time. However, the analysis did not encompass the post-treatment tumor necrosis rate in relation to recurrence risk and survival. As previously established, pretreatment AFP levels reflect tumor burden and aggressiveness. However, elevated AFP levels are observed in only approximately 30% of patients with HCC, limiting their diagnostic utility [30]. Consequently, greater emphasis is placed on their prognostic value. In the present study, univariate and multivariate Cox regression analyses did not identify AFP levels as a prognostic factor. Although a significant post-treatment reduction in AFP levels is associated with favorable outcomes, the 2024 edition of the Chinese guidelines for HCC established an AFP cutoff of 400 ng/mL and identified AFP levels ≥400 ng/mL as an independent prognostic factor for recurrence [31]. The low number of patients presenting with AFP levels above this threshold may account for the failure of our study in identifying AFP as an independent prognostic factor.

MVI is a well-defined independent prognostic factor for HCC recurrence. It is specifically characterized by the presence of microscopic nests of tumor cells within endothelial-lined vessels, including the portal vein and its branches. Consistent with this evidence, in the present study, MVI was demonstrated to be an independent prognostic factor following LT, aligning with tumor morphology as a tumor aggressiveness indicator. For the group of patients who remained beyond the MC following DS and thus experience a high recurrence risk, strategies, including early post-LT steroid withdrawal, steroid-free immunosuppressive regimens, calcineurin inhibitor reduction, and shortened follow-up intervals, may be recommended [32]. This study yielded several robust findings. It provided pooled estimates for survival and recurrence following DS and LDLT. Currently, no pooled analysis has been conducted regarding DS outcomes in patients undergoing LRT with DS-TACE. We observed successful DS in >50% of the patients and a treatment response in >90%. At the same time, our results eliminated certain limitations for patients who achieved successful DS but for whom LT was not previously considered an option under the accepted criteria. It provided clinical validation regarding the use of MC in the setting of DS for LDLT. LT should be considered for all patients with HCC following successful DS within the established transplantability criteria. Whether LDLT should be performed on patients who remain beyond the MC following DS remains debated. For patients who respond to DS therapy but remain beyond the MC, this approach provides greater OS and RFS benefits than other nontransplant strategies for LDLT-eligible recipients. A study involving 74 patients demonstrated that LT performed following successful and effective DS of tumors initially exceeding the MC caused improved OS and event-free survival compared with nontransplant strategies [4]. Patients who remained beyond the MC after downstaging require a distinct interpretation. İn our analysis, 3- and 5-year RFS estimates were both 40.9%, and the adjusted recurrence HR was 4.133 relative to patients initially within the MC. The OS curve was also numerically lower, but neither the global log-rank test nor the adjusted OS model reached statistical significance. Considering that DS serves as a prognostic tool and a means for identifying a patient subgroup with favorable tumor biology, predictive of better outcomes post-transplantation, expanding the morphological criteria for DS has become an attractive alternative. In this context, tumor progression and increasing AFP levels despite LRT reflect more aggressive tumors associated with a risk of HCC recurrence following LT. A defined observation period is essential for assessing tumor response and changes in AFP levels before LT. A large-scale multicenter randomized prospective study is warranted to identify treatment failure-associated factors, thereby establishing inclusion criteria for DS in the context of LDLT. This first multicenter study on DS using a uniform protocol exhibited excellent 5-year post-LDLT survival and a low HCC recurrence rate. These findings supported expanding priority access to LDLT for HCC recipients who respond to DS. These findings highlight the concept of a treatment hierarchy, suggesting that treatment efficacy rather than tumor stage alone should determine patient management. In the near future, the widespread adoption of novel systemic agents could further improve the DS potential of therapies, increase the number of patients with intermediate-stage HCC likely to benefit from LT, and alter current principles regarding treatment hierarchy. This study revealed excellent post-LDLT OS and RFS following DS, primarily achieved via DSM-TACE, under a uniform protocol. The DS strategy using DSM-TACE should be considered an option for selecting a subgroup of patients, specifically those exceeding the MC but possessing favorable tumor biology, for LT based on their response to LRT, rather than solely relying on expanded criteria. Current recommendations regarding the benefits of DS are based on small-scale noncomparative retrospective studies with short follow-up durations. By contrast, our study reported comparative long-term follow-up results involving only living-donor liver transplant recipients. Our findings are highly significant in terms of post-transplant survival outcomes following successful DS to within the MC, independent of organ allocation principles. This significance is further emphasized when considering the identification of patients who will truly benefit from transplantation. Our findings revealed similarly satisfactory survival rates in patients who were successfully downstaged from DS to the MC using DSM-TACE and in patients who were initially in the MC. Furthermore, employing DS should be encouraged even in recipients who were beyond the MC following DS. The present study demonstrates that the proportional-hazards analysis further qualifies the beyond MC result. The contrast was time-dependent and all recurrences occurred within 26 months; its HR therefore represents an average effect concentrated in the early post-transplant period rather than a constant long-term hazard ratio. The timing sensitivity analysis produced concordant estimates, supporting the direction of the association. A further strength is the long follow-up: the reverse Kaplan–Meier estimate was 66 months, and 3 and 5 year survival estimates could be reported.

In particular, it may enhance donor selection suitability for every patient who responds to the treatment.

This study had some limitations, including a substantial heterogeneity regarding the indications for LRT in patients with HCC falling outside standard criteria, as well as the inclusion and exclusion criteria used for selecting patients with HCC for DS. This study did not account for differences across various LRT modalities. DS was specifically administered as DSM-TACE, excluding other treatments, such as radiotherapy, systemic therapy, immunotherapy, and TARE. This study centered on patients with HCC who were selected for DS and fell outside the MC; no standardized DS protocol was established for this cohort. The retrospective nature of this study, alongside its small sample size, could lead to selection bias. Regarding OS benefit, performing an ITT analysis comparing patients who underwent transplantation following DS with those who did not may have been preferable.

## 5. Conclusions

To the best of our knowledge, no observational studies to date have used DSM-TACE for DS in patients undergoing LDLT for intermediate- or advanced-stage HCC. Our findings demonstrated that DSM-TACE represents a safe and effective DS method, yielding acceptable OS and RFS in patients with cirrhotic HCC and liver dysfunction. Furthermore, compared with other treatments, we observed a notable survival rate in patients who were initially beyond the MC but underwent DS. The survival rates in this group were comparable to those of patients who were initially within the MC and those who fell within the MC following DS. When considering DSM-TACE for DS in patients beyond the MC, the survival advantage gained by this group should be weighed against the risk of waitlist mortality. Following DS, macrovascular invasion was the sole independent prognostic factor for post-transplant recurrence across all groups. A certain response level to LRT could function as a criterion for LDLT, serving as a method for selecting patients with favorable biological characteristics.

## Figures and Tables

**Table 1:** Demographics and clinical-pathological characteristics of HCC patients.

Gender	Male	83 (79.0)
	Female	22 (21.0)
Age Median [IQR]		66.9 [59.8–71.0]
BMI Median [IQR]		26 [23–29]
Diagnosis n (%)	HBV	58 (55.2)
	HCV	14 (13.3)
	Cryptogenic	16 (15.2)
	Nash	8 (7.6)
	Ethanol	3 (2.9)
	Autoimmune	2 (1.9)
	Budd-Chiari	1 (1.0)
	Cholestasis	1 (1.0)
	non-cirrhosis	1 (1.0)
	Wilson	1 (1.0)
Milan n (%)	within Milan(initially)	65 (61.9)
	Beyond Milan after downstaging	17 (16.2)
	within Milan after downstaging	23 (21.9)
Child–Pugh grade n (%)	A	58 (55.2)
	B	40 (38.1)
	C	7 (6.7)
MELD Median [IQR]		11 [8–16]
AFP Median [IQR]		9 [4–45]
Tumor size(Max.mm) Median [IQR]		25 [17–40]
Number of HCC lesions Median [IQR]		1 [1–2]
Locoregional treatment n (%)		40 (38.1)
Live n (%)		81 (77.1)
Tumor recurrence n (%)		16 (15.2)
Tumor differentiation n (%)	early	36 (34.3)
	poorly	21 (20.0)
	intermediate	48 (45.7)
Microvascular invasion n (%)		34 (32.4)
Mortality n (%)		24 (22.9)
Follow-up time Median [IQR]		55 [28–77]
Graft-to-recipient weight ratio Median [IQR]		1 [0.9–1.1]
Cold ischemia time (graft) Median [IQR]		100 [80–115]
Downstaging therapy	Non-treatment	65 (61.9)
	TACE	40 (38.1)
Response to LRT	Progression	3 (7.5)
	Regression	23 (57.5)
	Response	2 (5.0)
	Stabil	12 (30.0)

Nash, non-alcoholic steatohepatitis; MELD, Model for End-Stage Liver Disease; AFP, Alpha Feto Protein.

**Table 2:** Cox regression analyses for overall survival.

Variable/Category	Univariable Conventional Cox Regression		Multivariable Firth-Penalized Cox Regression	
	HR (95% CI)	*p*	HR (95% Profile CI)	*p*
AFP (per 1 ng/mL)	0.999 (0.996–1.002)	0.487	—	—
MELD score (per 1 point)	1.003 (0.941–1.069)	0.920	—	—
Microvascular invasion				
Absent (reference)	1.000 (reference)	—	1.000 (reference)	—
Present	1.727 (0.773–3.855)	0.183	1.462 (0.624–3.355)	0.375
Tumor size (per 1 mm)	1.013 (0.992–1.034)	0.238	—	—
Number of HCC lesions (per lesion)	1.047 (0.903–1.213)	0.544	—	—
Child–Pugh class (overall)	—	0.437	—	—
A (reference)	1.000 (reference)	—	—	—
B	1.068 (0.450–2.536)	0.881	—	—
C	2.481 (0.699–8.804)	0.160	—	—
Milan category (overall)	—	0.160	—	0.249
Within Milan (reference)	1.000 (reference)	—	1.000 (reference)	—
Outside Milan after downstaging	2.459 (0.978–6.184)	0.056	2.214 (0.818–5.603)	0.113
Within Milan after downstaging	0.905 (0.295–2.777)	0.862	0.959 (0.290–2.607)	0.938

HR, hazard ratio; CI, confidence interval; MELD, Model for End-Stage Liver Disease. Univariable estimates reproduce the conventional Cox analyses in the original report. Reference categories are shown explicitly. For multi-level categorical variables, the overall *p*-value is shown on the factor row and level-specific *p*-values compare each level with the stated reference. In the univariable column, overall factor *p*-values are likelihood-ratio tests and level-specific *p*-values are Wald tests. The multivariable model was restricted to Milan category (2 df) and microvascular invasion (1 df) and fitted with Firth-penalized partial likelihood using Breslow handling of ties; 95% profile penalized-likelihood CIs and penalized likelihood-ratio *p*-values are reported.

**Table 3:** Cox regression analyses for recurrence-free survival.

Variable/Category	Univariable Conventional Cox Regression		Multivariable Firth-Penalized Cox Regression	
	HR (95% CI)	*p*	HR (95% Profile CI)	*p*
AFP (per 1 ng/mL)	1.000 (0.998–1.002)	0.919	—	—
MELD score (per 1 point)	0.909 (0.816–1.013)	0.085	—	—
Microvascular invasion				
Absent (reference)	1.000 (reference)	—	1.000 (reference)	—
Present	9.641 (2.746–33,848)	<0.001	5.990 (1.924–24,048)	0.001
Tumor size (per 1 mm)	1.030 (1.009–1.052)	0.006	—	—
Number of HCC lesions (per lesion)	1.147 (1.021–1.287)	0.020	—	—
Child–Pugh class (overall)	—	0.055	—	—
A (reference)	1.000 (reference)	—	—	—
B	0.240 (0.054–1.074)	0.062	—	—
C	1.661 (0.371–7.428)	0.507	—	—
Milan category (overall)	—	0.002	**—**	0.041
Within Milan (reference)	1.000 (reference)	—	1.000 (reference)	—
Outside Milan after downstaging	7.932 (2.582–24,373)	<0.001	4.133 (1.377–13,564)	0.012
Within Milan after downstaging	2.047 (0.489–8.573)	0.327	1.872 (0.433–7.056)	0.375

HR, hazard ratio; CI, confidence interval; MELD, Model for End-Stage Liver Disease. Univariable estimates reproduce the conventional Cox analyses in the original report. Reference categories are shown explicitly. For multi-level categorical variables, the overall *p*-value is shown on the factor row and level-specific *p*-values compare each level with the stated reference. In the univariable column, overall factor *p*-values are likelihood-ratio tests and level-specific *p*-values are Wald tests. The multivariable model was restricted to Milan category (2 df) and microvascular invasion (1 df) and fitted with Firth-penalized partial likelihood using Breslow handling of ties; 95% profile penalized-likelihood CIs and penalized likelihood-ratio *p*-values are reported. Scaled Schoenfeld-residual diagnostics in the corresponding conventional Cox model indicated nonproportionality for the outside Milan after downstaging contrast (*p* = 0.021); this HR should be interpreted as a follow-up-weighted average.

## Data Availability

All data generated or analyzed during this study are included in this article. Further enquiries can be directed to the corresponding author.

## Abbreviations

DSM-TACE	degradable starch microspheres with transcatheter arterial chemoembolization
HCC	hepatocellular carcinoma
Nash	non-alcoholic steatohepatitis
Meld	model for end-stage liver disease
AFP	alpha feto protein
BMI	body mass index
MVI	microvascular invasion
OS	overall survival
RFS	recurrence-free survival
MC	Milan criteria
LRT	locoregional therapy
TARE	transarterial radioembolization
LDLT	living-donor liver transplantation
DS	downstaging
mRECIST	response evaluation criteria in solid tumors
VEGF	vascular endothelial growth factor
